# 40 Hz Blue LED Relieves the Gamma Oscillations Changes Caused by Traumatic Brain Injury in Rat

**DOI:** 10.3389/fneur.2022.882991

**Published:** 2022-06-21

**Authors:** Xiaoyu Yang, Xuepei Li, Yikai Yuan, Tong Sun, Jingguo Yang, Bo Deng, Hang Yu, Anliang Gao, Junwen Guan

**Affiliations:** ^1^Department of Neurosurgery, West China Hospital, Sichuan University, Chengdu, China; ^2^Medical Simulation Center, Chengdu First People's Hospital, Chengdu, China; ^3^College of Electronic Engineering (College of Meteorological Observation), Chengdu University of Information Technology, Chengdu, China; ^4^Department of Neurosurgery, The Second Affiliated Hospital of Chengdu Medical College, China National Nuclear Corporation 416 Hospital, Chengdu, China

**Keywords:** traumatic brain injury, gamma oscillations, LED, rat, electroencephalogram

## Abstract

**Background:**

Photobiomodulation (PBM) using low-level light-emitting diodes (LEDs) can be rapidly applied to various neurological disorders safely and non-invasively.

**Materials and Methods:**

Forty-eight rats were involved in this study. The traumatic brain injury (TBI) model of rat was set up by a controlled cortical impact (CCI) injury. An 8-channel cortex electrode EEG was fixed to two hemispheres, and gamma oscillations were extracted according to each electrode. A 40 hz blue LED stimulation was set at four points of the frontal and parietal regions for 60 s each, six times per day for 1 week. Modified Neurologic Severity Scores (mNSS) were used to evaluate the level of neurological function.

**Results:**

In the right-side TBI model, the gamma oscillation decreased in electrodes Fp2, T4, C4, and O2; but significantly increased after 1 week of 40 hz Blue LED intervention. In the left-side TBI model, the gamma oscillation decreased in electrodes Fp1, T3, C3, and O1; and similarly increased after 1 week of 40 hz Blue LED intervention. Both left and right side TBI rats performed significantly better in mNSS after 40 hz Blue LED intervention.

**Conclusion:**

TBI causes the decrease of gamma oscillations on the injured side of the brain of rats. The 40 hz Blue LED therapy could relieve the gamma oscillation changes caused by TBI and improve the prognosis of TBI.

## Graphical Abstract

Experimental grouping and process. 
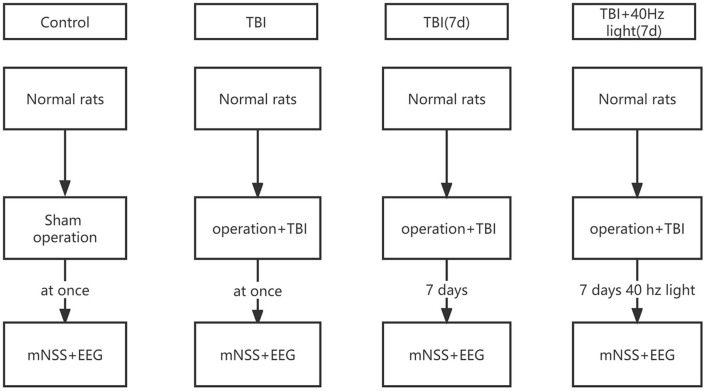

## Introduction

The invention of the electroencephalogram (EEG) facilitates the observation of brain electrical activity (1, 2); the most common frequency band in EEG is 8–12 Hz, known as α-rhythm, and there are faster oscillations where the frequency is between 30 and 80 Hz, named gamma oscillations (3). Gamma oscillations were first discovered by Adrian (4) who reported that the oscillations were induced by odorous substances. Since, studies have demonstrated that they could be induced by other stimuli and be presented in various structures in the human brain, with different functional connections, such as learning, sensory, consciousness, and motor functions (5–8).

More than 50 million people suffer TBIs each year worldwide, and it is a major cause of death and disability for children and adults in all countries, leading to a great amount of burden upon society (9). Deficiencies in TBI research need to be emphasized to figure out solutions to reduce societal costs of TBI. Although many works have investigated the relationship between changes in gamma oscillations and abnormality in the nervous system (10–12), the interplay between gamma oscillations and TBI has yet to be confirmed. In general, changes in brain electrical activity occur after TBI (13, 14). A study showed that subjects with TBI display disturbed gamma synchrony activity (15), but they did not propose the degree of changes. We aim to determine how gamma oscillations change in TBI models of different degrees of injury.

A small proportion of severe TBIs will remain in a coma or vegetative state (16), and many TBI patients are likely to exhibit cognitive function decline and motor dysfunction (17). Rehabilitation for patients with TBI is a complex process, the possible therapy includes right median nerve stimulation (16), but the therapeutic benefits need further verification. Bain PBM therapy is an innovative and effective treatment used in various neurological diseases such as TBI, Alzheimer's disease, and age-related cognitive decline (18, 19). This non-invasive therapy could produce treatment effects without causing adverse influence. Non-invasive 40 Hz light could stimulate elevation of gamma oscillations (16, 20). We further observe the interplay between gamma oscillations changes and try to observe prognosis in TBI models.

Understanding how gamma oscillations changes after TBI has implications for further intervention. Our purpose is to determine how gamma oscillations change following TBI and examine whether non-invasive 40 Hz light could be a possible intervention to improve the prognosis in TBI models.

## Materials and Methods

### Animals

A total of 48 two-month-old male Sprague-Dawley rats (200–225 g,) were obtained from the Laboratory Animal Center of Sichuan University, and free access was given to food and water throughout the study. All the rats scored 0 in mNSS before the experiment and there was no statistical difference. Animals were randomly divided into left hemisphere TBI and right hemisphere TBI and each was divided into four subgroups: control (rats received sham operation); TBI (rats received TBI induced by an impact device and immediate 8-channel cortex electrodes EEG detection after injury); TBI (7d) (8-channel cortex electrodes EEG was obtained seven days after TBI); TBI+40 Hz light (7d) (rats received 40 hz Blue LED intervention for consecutive seven days after TBI). Each subgroup consisted of six rats.

### TBI Model

The traumatic brain injury (TBI) model of the rats was set up by a controlled cortical impact (CCI) injury (21). Rats were anesthetized with 5% isoflurane and then maintained with 2% isoflurane for the duration of the procedure. After the operative region was shaved, the animal was positioned in a stereotactic frame. Then, the skin above the skull was prepped with bated iodine solution, and the skull was exposed through a small skin incision made by a sterile scalpel blade. An area about 5 mm in diameter was exposed with the use of a dental drill, and the center of the area is 2.7 mm beside the midline and 3 mm in front of the lambda suture. The dura was kept intact during the operation. The exposed cortex was subjected to CCI. The position of the impactor was is 1.2 mm beside the midline and 1.5 mm in front of the lambda suture. CCI was produced carefully in the center above the craniotomy area, using an electromagnetic CCI device with a 3.0 mm diameter impactor rod-tip, hitting the cortex at a velocity of 4.0 m/s with an impact depth of 2.5 mm and impact time of 50 ms to produce injury within the left or right parietal cortex. After the CCI procedure, bone wax was applied to the exposed surface of the dura and skull surface. The skin incision was sutured, followed by the application of bated iodine solution over the sutured area. The same surgical procedures were done to the sham injury control rats except for the CCI procedures. Animals with TBI were kept warm during the experiments.

### Gamma Data Acquisition

**Figure 1:**
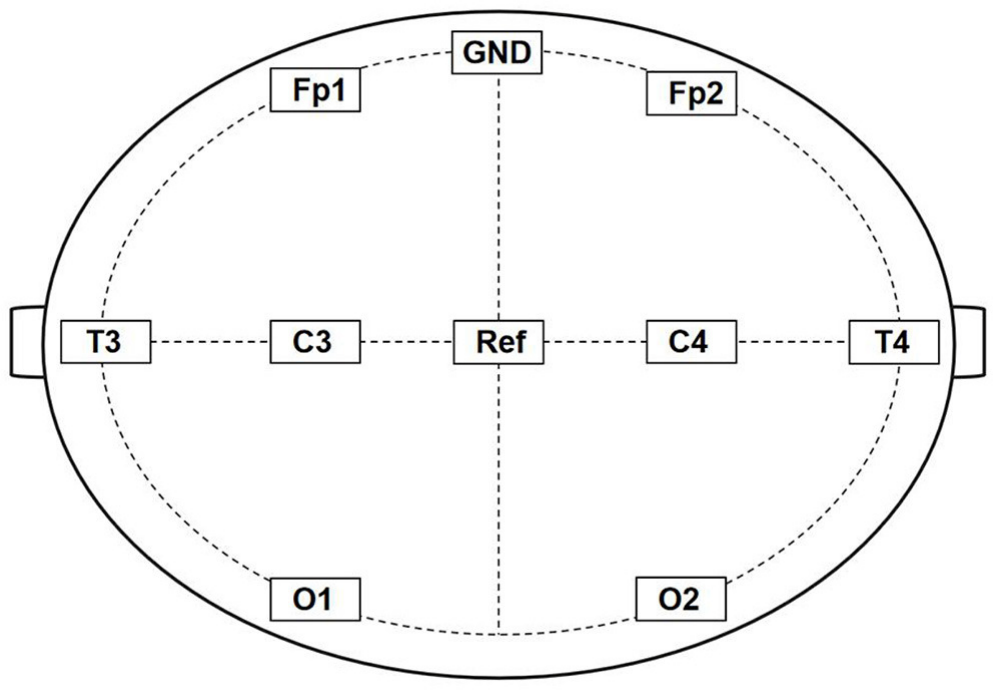
Schematic diagram of 8-channel cortex electrodes EEG. Fp1, T3, C3, and O1 are fixed to the frontal, temporal, parietal, and occipital lobes of the left hemisphere, whereas Fp2, T4, C4, and O2 are fixed to the frontal, temporal, parietal, and occipital lobe of the right hemisphere, respectively. GND was connected to an electrical ground and Ref is the Reference electrode.

All animals were placed stably in a stereotaxic apparatus to acquire the gamma data using an 8-channel cortex electrodes EEG (Fp1, Fp2, T3, T4, C3, C4, O1, and O2). Fp1, T3, C3, and O1 are fixed to the frontal, temporal, parietal, and occipital lobes of the left hemisphere, whereas Fp2, T4, C4, and O2 are fixed to the frontal, temporal, parietal, and occipital lobe of the right hemisphere, respectively (Figure 1). We recorded the gamma oscillations power of control and TBI groups after CCI or the sham operation, respectively. Similarly, we recorded the gamma oscillations power 1 week after TBI.

#### 40 Hz Blue Light-Emitting Diode Therapy Protocol

Rats were submitted to 40 hz Blue LED at a 473 nm wavelength, with 0.2 W LED power, 70-mW/cm^2^ power density, 10-J/cm^2^ energy density, 13-mm spot size, and 40 J/cm^2^ per treatment. At short wavelengths, absorption occurs predominantly by chromophores such as melanin and hemoglobin.

In order to avoid skin burning, the power density of 70 mW/cm^2^ was considered safe. The wavelength (~473 nm) is characterized by a relatively low energy density (40 J/cm^2^) for therapeutic uses. The application of LED irradiation will be in four points in the frontal and parietal regions for 60 s at each point (240 s total/session) six times per day during a week.

#### Modified Neurologic Severity Scores

The mNSS was used to evaluate the level of neurological function of TBI rats. The neurological severity score is a composite of motor, sensor, balance, and reflex which could reflect the level of neurological function of rats (22–24). The higher the score, the more severe the injury (Table 1).

**Table 1:** Modified neurological severity score (mNSS).

**Tests**	**Points**
**Motor tests** Raising rat by the tail (normal = 0; maximum = 3)	6 3
Flexion of forelimb	1
Flexion of hindlimb	1
Head moved >10° to vertical axis within 30 s	1
Placing rat on the floor (normal = 0; maximum = 3)	3
Normal walk	0
Inability to walk straight	1
Circling toward the paretic side	2
Fall down to the paretic side	3
**Sensory tests**	2
Placing test (visual and tactile test)	1
Proprioceptive test (deep sensation, pushing the paw against the table edge to stimulate limb muscles)	2
**Beam balance tests** (normal = 0; maximum = 6)	6
Balances with steady posture	0
Grasps side of beam	1
Hugs the beam and one limb falls down from the beam	2
Hugs the beam and two limbs fall down from the beam, or spins on beam (>60 s)	3
Attempts to balance on the beam but falls off (>40 s)	4
Attempts to balance on the beam but falls off (>20 s)	5
Falls off: No attempt to balance or hang on to the beam (<20 s)	6
**Reflexes absent and abnormal movements**	4
Pinna reflex (head shake when touching the auditory meatus)	1
Corneal reflex (eye blink when lightly touching the cornea with cotton)	1
Startle reflex (motor response to a brief noise from snapping a clipboard paper	1
Seizures, myoclonus, myodystony	1
**Maximum points**	18

#### Statistical Analysis

All the data was described as mean ± SEM. Statistical analysis was performed with SPSS for Windows, version 19.0. The differences among multiple groups were assessed using a one-way analysis of variance (ANOVA) with a significance level of *p* < 0.05. *Post-hoc* comparisons between groups were further detected using the least significant difference (LSD) method. Sphericity test used methods KMO and Bartlett's Test. All statistical analysis was done by a professional statistician.

## Result

**Figure 2:**
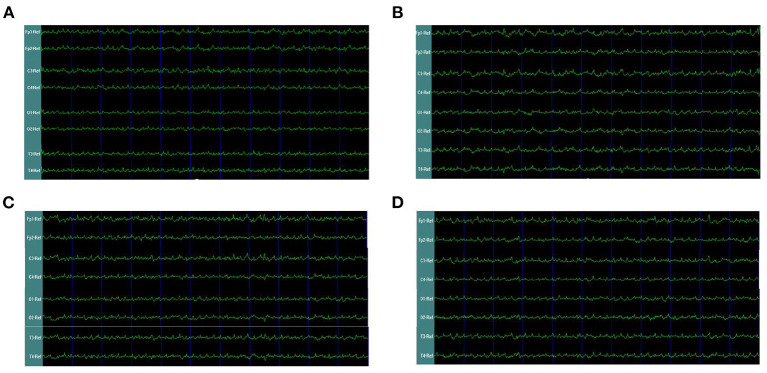
EEG waveforms of right-side TBI in rats. **(A)** EEG waveforms of control subgroup **(B)** EEG waveforms recorded immediately after TBI **(C)** EEG waveforms of 7 days after TBI without treatments **(D)** EEG waveforms of TBI after 7-days treatment of 40 hz Blue LED.

**Figure 3:**
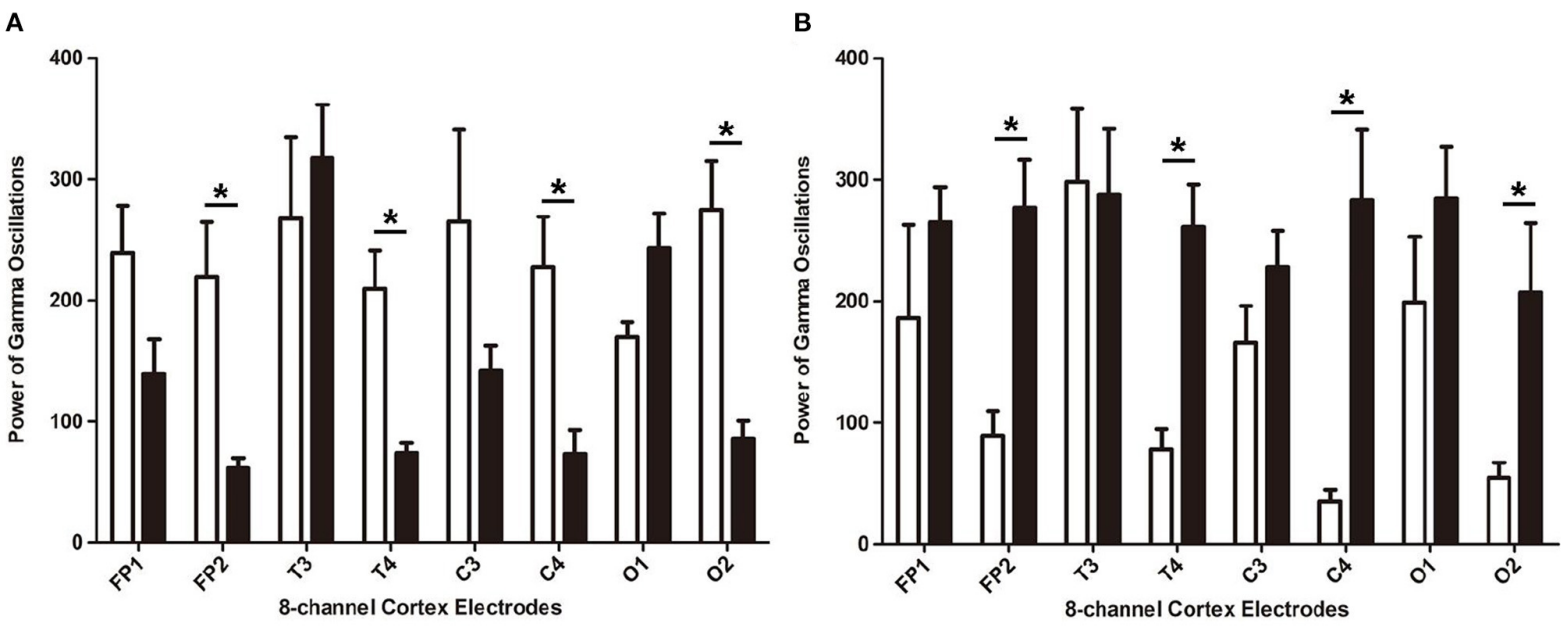
The power of gamma oscillations of right-side TBI group in different electrodes. **(A)** Power of gamma oscillations of rat before and after TBI. The white bar represents the control group (a), and the black bar represents TBI (R, 7d) group. **(B)** Power of gamma oscillations of TBI with and without treatment of 40 hz Blue LED. The white bar represents TBI (R, 7d), and the black bar represents TBI (R)+40 hz light (7d). *P*-values correspond to one-way ANOVA. Data are expressed as mean±SEM. *P < 0.05.

In the rats with moderate to severe TBI on the right side, the EEG pattern was shown in Figure 2. The gamma oscillation we recorded after TBI showed a significant decrease in cortex electrodes Fp2 (*P* = 0.002), T4 (*P* = 0.001), C4 (*P*= 0.009), and O2 (*P* = 0.001) (Figure 3A). After 1 week of 40 hz Blue LED intervention, the gamma oscillation of rats significantly increased in these cortex electrodes, Fp2 (*P* = 0.001), T4 (*P* < 0.001), C4 (*P* < 0.001), and O2 (*P* < 0.007), compared with the TBI (R,7d) group (Figure 3B, Table 2).

**Table 2:** Gamma oscillations of TBI (R, 7d) and TBI (R)+40 hz light (7d) rats.

**Cortex electrodes**	**TBI(R, 7d)** **(mean ± SEM)**	**TBI(R)+40 hz light (7d)** **(mean ± SEM)**	***P***
FP1	186.34 ± 187.46	265.09 ± 71.09	0.255
FP2	89.42 ± 49.66	276.87 ± 97.05	0.001*
T3	298.23 ± 147.36	288.08 ± 131.51	0.901
T4	77.94 ± 41.42	261.17 ± 85.56	<0.001*
C3	166 ± 73.68	227.98 ± 73.58	0.339
C4	35.52 ± 21.9	282.91 ± 142.41	<0.001*
O1	199.1 ± 132.53	284.27 ± 105.97	0.129
O2	54.79 ± 29.88	207.18 ± 139.84	0.007*

Absolute energy of gamma oscillations for each cortex electrode were calculated. P-values correspond to one-way ANOVA between TBI (R, 7d) and TBI (R)+40 hz light (7d) rats. Data are expressed as mean ± SEM. *P <0.05.

**Figure 4:**
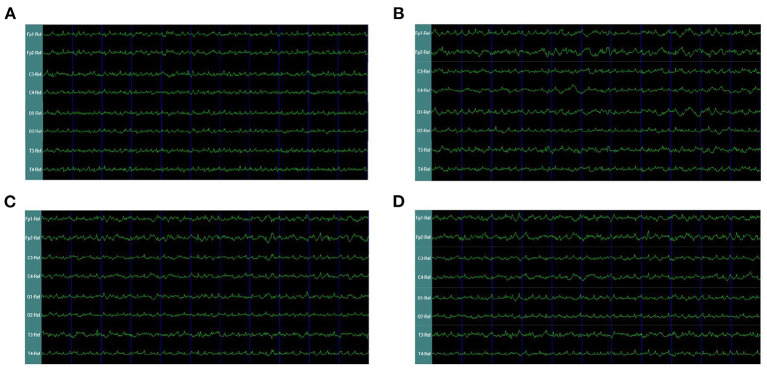
EEG waveforms of left-side TBI in rats. **(A)** EEG waveforms of control subgroup **(B)** EEG waveforms recorded immediately after TBI **(C)** EEG waveforms of 7 days after TBI without treatments **(D)** EEG waveforms of TBI after 7-days treatment of 40 hz Blue LED.

**Figure 5:**
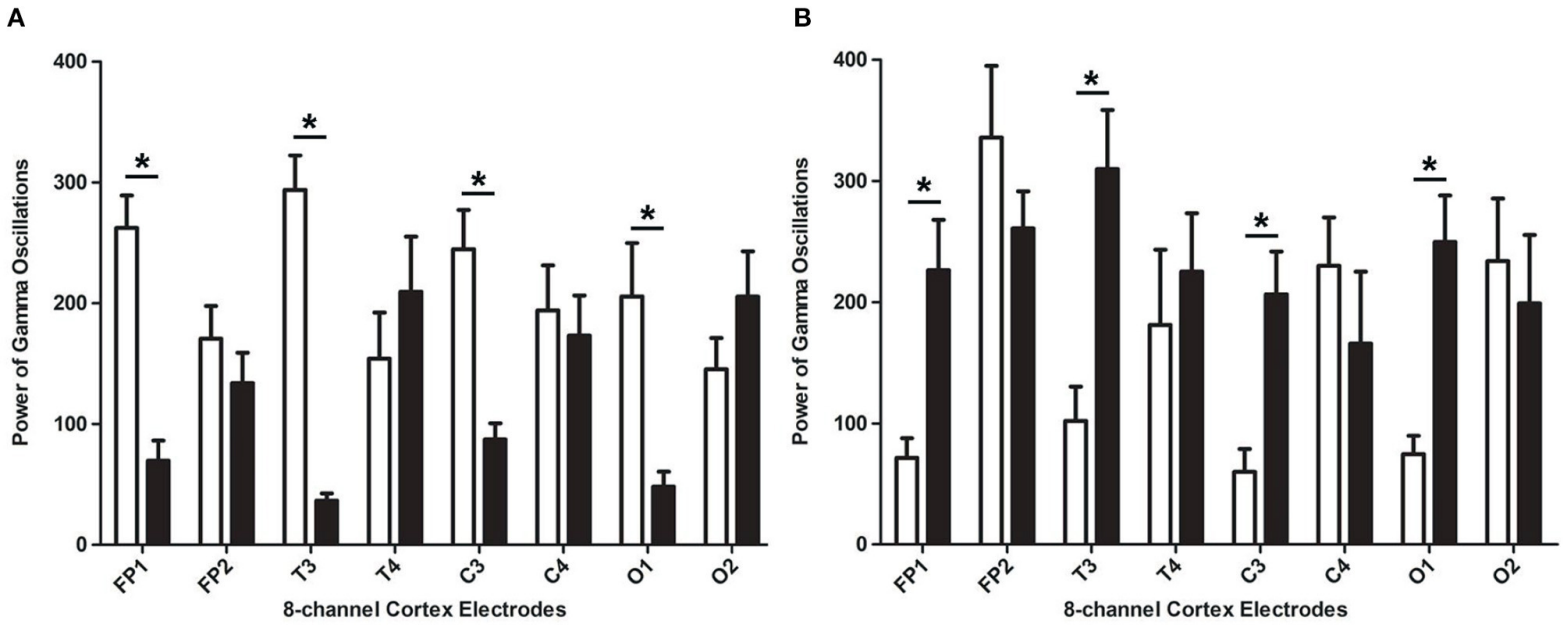
The absolute energy of gamma oscillations of left-side TBI group in different electrodes. **(A)** Absolute energy of gamma oscillations of rat before and after TBI. The white bar represents control group (b), and the black bar represents TBI (L, 7d). **(B)** Absolute energy of gamma oscillations of TBI with and without 7-days treatment of 40 hz Blue LED. The white bar represents TBI (L, 7d), and the black bar represents TBI (L)+40 hz light (7d). *P*-values correspond to one-way ANOVA. Data are expressed as mean ± SEM. *P < 0.05.

In our second phase of the research, the rat model of left side TBI was built. The EEG pattern is shown in Figure 4. We found that the gamma oscillation of rats with left TBI significantly decreased in cortex electrodes Fp1 (*P* < 0.001), T3 (*P* < 0.001), C3 (*P* < 0.001), and O1 (*P* = 0.002) (Figure 5A, Table 3). Similarly, after receiving 1 week of 40 hz Blue LED intervention, the gamma oscillation of rats significantly increased in these cortex electrodes Fp1 (*P* = 0.001), T3 (*P* < 0.001), C3 (*P* = 0.001), and O1 (*P* = 0.001), comparing with the TBI(L,7d) group (Figure 5B, Table 4) and this result was completely consistent with that of right side TBI rats.

**Table 3:** Gamma oscillations of control (b) and TBI (L) rats.

**Cortex electrodes**	**Control (b)** **(mean ± SEM)**	**TBI(L)** **(mean ± SEM)**	***P***
FP1	262.4 ± 65.79	69.83 ± 40.13	<0.001*
FP2	170.5 ± 66.83	133.88 ± 61.39	0.505
T3	293.91 ± 69.91	36.62 ± 14.49	<0.001*
T4	154.27 ± 93	209.36 ± 112.2	0.439
C3	244.59 ± 79.87	87.18 ± 32.8	<0.001*
C4	194.02 ± 91.45	172.97 ± 81.79	0.737
O1	205.63 ± 108.2	48.25 ± 30.03	0.002*
O2	145.3 ± 63.11	205.46 ± 91.76	0.351

Absolute energy of gamma oscillations for each cortex electrode was calculated. P-values correspond to one-way ANOVA between control (b) and TBI(L) rats. Data are expressed as mean ± SEM. *P <0.05.

**Table 4:** Gamma oscillations of TBI (L, 7d) and TBI (L)+40 hz light (7d) rats.

**Cortex Electrodes**	**TBI (L, 7d)** **(mean ± SEM)**	**TBI (L)+40 hz light (7d)** **(mean ± SEM)**	***P***
FP1	71.47 ± 39.83	226.33 ± 102.24	0.001*
FP2	335.79 ± 145.19	261.02 ± 74.83	0.181
T3	102.15 ± 69.02	309.79 ± 119.81	<0.001*
T4	181.31 ± 152.02	225.17 ± 118.35	0.536
C3	59.96 ± 45.88	206.56 ± 86.13	0.001*
C4	229.97 ± 97.93	165.67 ± 145.61	0.311
O1	74.53 ± 37.49	249.68 ± 93.98	0.001*
O2	233.83 ± 127.07	199.03 ± 138.2	0.587

Absolute energy of gamma oscillations for each cortex electrode was calculated. P-values correspond to one-way ANOVA between TBI (L, 7d) and TBI (L)+40 hz light (7d) rats. Data are expressed as mean ± SEM. *P <0.05.

**Figure 6:**
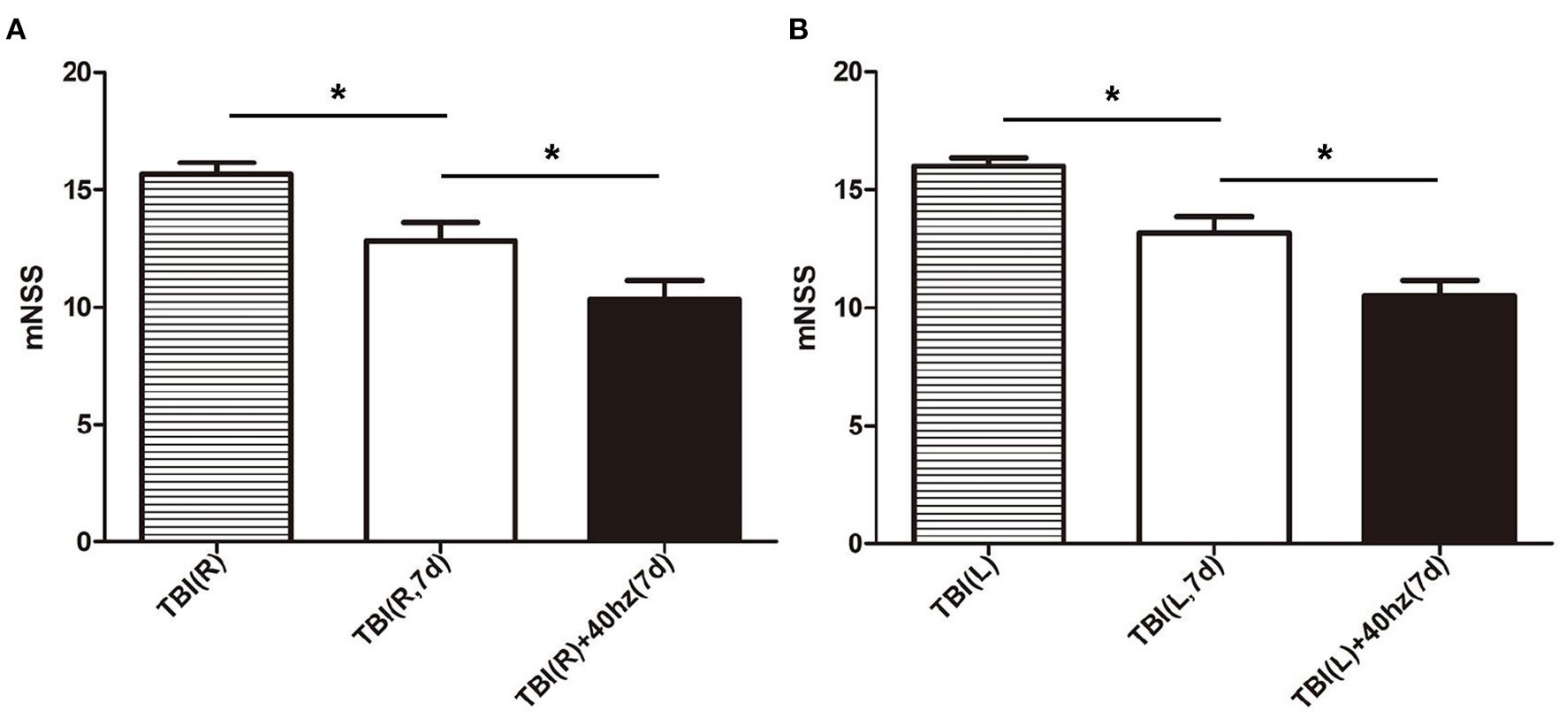
The mNSS in different subgroups. **(A)** The mNSS evaluated of right-side TBI group. The stripe, white and black bar represent for TBI (R), TBI (R, 7d) and TBI (R)+40 hz light (7d), respectively. **(B)** The mNSS evaluated of left-side TBI group. The stripe, white and black bar represent for TBI (L), TBI (L, 7d), and TBI(L)+40 hz light (7d), respectively. *P*-values correspond to one-way ANOVA. Data are expressed as mean ± SEM. *P < 0.05.

The rats that received 40 hz Blue LED invention performed significantly better in mNSS than the TBI rats 7 days after injury. This result appeared both in the right side TBI (*P* = 0.013) (Figure 6A) and left side TBI models (*P* = 0.004) (Figure 6B, Table 5).

**Table 5:** mNSS of TBI (7d) and TBI+40 hz light (7d) rats.

**TBI model**	**TBI** **(mean ± SEM)**	**TBI+40hz light** **(mean ± SEM)**	***P***
Right side	15.67 ± 1.21	12.83 ± 1.94	0.013*
Left side	16.00 ± 0.89	13.17 ± 1.72	0.004*

mNSS for two types of TBI models were calculated. P-values correspond to one-way ANOVA between TBI (7d) and TBI+40 hz light (7d). Data are expressed as mean ± SEM. *P <0.05.

## Discussion

Gamma oscillations play a pivotal role in multiple cognitive functions. They enable coordinated activity and communication of local assemblies, while abnormalities in gamma oscillations exist in different neurological and psychiatric diseases (25, 26). Increasing evidence shows that guiding or driving gamma oscillations are being investigated as a potential treatment paradigm for memory deficits due to Alzheimer's disease and other memory impairments (27, 28), but it has not been explored in TBI. Hannah et al. have reported that 40 Hz gamma oscillations may induce an overall neuroprotective response that recruits both neurons and microglia (11). In our study, we are trying to find out how TBI effects the gamma oscillations in rats, and if 40 hz Blue LED could be a non-invasive adjuvant therapy for TBI.

In our study, the cortex electrodes Fp1, T3, C3, and O1 were fixed to the frontal, temporal, parietal, and occipital lobe of the left hemisphere, and the cortex electrodes Fp2, T4, C4, and O2 were fixed to the frontal, temporal, parietal, and occipital lobe of the right hemisphere, respectively. In the first phase, we found that the rats with right side TBI showed a decrease in cortex electrodes Fp2, T4, C4, and O2, which corresponds to the injured side. This result coincides with several previous studies which have demonstrated that lateral fluid percussion TBI in rats resulted in decreased theta power (29, 30). After building the TBI model, the rats received 40 hz Blue LEDs for 60 s at each point during a week. Surprisingly, the result showed TBI+40 hz light (R-7d) subgroup had a significant increase in those cortex electrodes but remained at the same level in the Fp1, T3, C3, and O1 cortex electrodes. In other words, the 40 hz Blue LED intervention partly reverses the decline in gamma oscillations caused by TBI.

In order to verify the effectiveness and feasibility of 40 hz Blue LED therapy, we established the left brain injury rat model in the second stage. We built rat TBI model of the left side,'with all other conditions being completely the same. As expected theoretically, after the TBI in the left side, we recorded a decrease in cortex electrodes Fp1, C3, T3, and O1. This indicates that the gamma oscillation decreases only in the affected side of the TBI, but have no influence on the lobe of the healthy side. After the 40 hz Blue LED intervention for a week, the gamma oscillations in cortex electrodes Fp1, C3, T3, and O1 were increased significantly, compared to the TBI(L, 7d) group. The results of these two phases strongly suggest that 40 hz Blue LED therapy could effectively reverse the gamma oscillations decrease in the injured cerebral cortex.

Many studies have reported that continuous low-frequency stimulation could significantly improve spatial learning in injured animals (31, 32). The effects of PBM therapy have also received increasing attention on multiple diseases, especially neurological disorders such as Alzheimer's and Parkinson's (26, 33). However, relatively few studies focus on the PBM therapy for TBI. In our study, the rats received 40 hz Blue LED therapy performed significantly better in mNSS than the TBI rats 7 days after injury; we could infer that 40 hz Blue LED therapy could improve the prognosis of different types of TBI, and possibly by increasing the gamma oscillations to repair the neurological function. These results are is analogous to some research groups that focused on the PBM therapy in TBI. Oron et al., for example, examined the effects of low-level laser therapy for TBI mice. A closed-head injury was induced by a weight-drop device. They found that TBI mice treated with laser showed better outcomes in neurobehavioral function (34). Khuman et al. demonstrated that low-level laser light therapy could improve cognitive function in CCI mice (35). The underlying mechanism could be explained in two phases. In the first phase, optogenetics induce or enhance the gamma oscillations. It has been demonstrated that by stimulating the optogenetic activator channelrhodopsin-2 (ChR2), expressed either in excitatory neurons of CA3 or CA1 or in stellate cells and interneurons in the mEC, local gamma oscillations can be induced in each of these areas (36–38). In the second phase, induced gamma oscillations in the rat highlight the intersection both in memory and locomotion mediating well-timed interactions within and across neuronal networks (39, 40). Conclusively, our research indicates that 40 hz Blue LED intervention may be an effective and novel neuromodulatory therapy for the treatment of persistent cognitive deficits following TBI.

## Conclusion

TBI causes a decrease of gamma oscillations in the injured brain side of rats. It appears 40 hz Blue LED therapy could relieve the gamma oscillation change caused by TBI and improve the prognosis of TBI which have the potential to become an effective and novel neuromodulatory therapy for the treatment of persistent cognitive deficits following TBI.

## Data Availability Statement

The raw data supporting the conclusions of this article will be made available by the authors, without undue reservation.

## Ethics Statement

The animal study was reviewed and approved by College of Basic Medicine and Forensic Medicine, Sichuan University.

## Author Contributions

XY: study concept and design, animal experiments, analysis, and interpretation of data. XL: design and interpretation data. BD, YY, and TS: animal experiments. JY and HY: statistical analysis. AG and JG: study supervision. All authors contributed to the article and approved the submitted version.

## Funding

This study was funded by the 1.3.5 project for disciplines of excellence-Clinical Research Incubation Project, West China Hospital, Sichuan University (21HXFH013), the National Natural Science Foundation of China (31371180), and the Key Technology Research and Development Program of Science and Technology of Sichuan Province (2015SZ0193).

## Conflict of Interest

The authors declare that the research was conducted in the absence of any commercial or financial relationships that could be construed as a potential conflict ofinterest.

## Publisher's Note

All claims expressed in this article are solely those of the authors and do not necessarily represent those of their affiliated organizations, or those of the publisher, the editors and the reviewers. Any product that may be evaluated in this article, or claim that may be made by its manufacturer, is not guaranteed or endorsed by the publisher.
